# Consensus on Integrated Care for Older People Among Dutch Experts: A Delphi Study

**DOI:** 10.5334/ijic.5682

**Published:** 2021-12-08

**Authors:** Anam Ahmed, Maria ETC van den Muijsenbergh, Hubertus JM Vrijhoef

**Affiliations:** 1 Panaxea b.v., Amsterdam, The Netherlands; 2 Department of Primary and Community Care, Radboud University Medical Centre, Nijmegen, the Netherlands; 3 Department of Prevention and Care, Pharos, Utrecht, The Netherlands; 4 Department of Patient and Care, Maastricht University Medical Centre, Maastricht, The Netherlands

**Keywords:** integrated care, older people, Delphi, realist research

## Abstract

**Introduction:**

In a previous rapid realist review (RRR), an initial programme theory (PT) was established giving insight into the interrelatedness of context items, mechanisms, programme-activities, and outcomes that influence integrated care programmes (ICPs) for community-dwelling frail older people. As ICPs need to be tailored to their local setting, the objective of this study is to assess consensus on the relevance of the items identified in the RRR for the Dutch setting, and refine the PT, where appropriate.

**Methods:**

A two-round e-Delphi study was carried out among Dutch experts to determine the relevance of 71 items.

**Results:**

Consensus on relevance was reached on 57 out of 71 items (80%). Items added to refine the PT included: increasing number of older people, decreasing access to hospital beds, well-designed ICP implementation processes, case management, having a clear portfolio of patients, the role of the government, aligning existing health and social care systems, management and monitoring of care activities, strong relationship between older person and healthcare providers (HCP), and providing continuous feedback to HCPs.

**Conclusion and discussion:**

The initial PT was refined for the Dutch setting. Items on which no consensus was found, need to be further investigated on the reason behind it.

## Introduction

Aging is irrevocably accompanied by the loss of physical, mental, and social strength and capabilities [1,2,3]. To address the diverse needs of older people for care and support, integrated care programmes (ICPs) are recommended. ICPs aim to provide a continuum of care for older people, where professionals in different domains cooperate and coordinate care taking into account the often complex care needs, and the individual preferences of older people within a broad range of (health and social) services over an extensive timeframe [4,5].

Notwithstanding the conceptual attractiveness of ICPs, the scientific literature shows heterogeneity in their outcomes [6,7,8,9].

### Care for older people in the Netherlands

The Dutch government aims to help people grow old independently in their trusted environment among others by facilitating better support and care at home, support to informal caregivers and volunteers, and more suitable housing for older people at the mean time saving on costs for institutional care [10]. General practitioners (GPs) and their specialised ‘primary care assistant practitioners’ are considered the main providers of complex care for older people. GPs, specialists in geriatric medicine and social geriatricians are partners in geriatric care who, together with pharmacists and home care providers, indicate what structure and resources are required to provide good care [11]. Primary care assistant practitioners are regarded as essential players in identifying care needs of patients, organizing care and coordinating primary care [12].

### Rapid Realist Review

This study is part of a larger study, commissioned by the National Health Care Institute. In the first part of this study, an international rapid realist review (RRR) [13] was conducted with the objective to provide insight into the relationships between the *context (C)* (wider external items) in which ICPs for community-dwelling older people are applied, the *mechanisms (M)* (enablers, underlying entities, processes, or structures) by which the ICPs (do not) work, and the *outcomes* (O) (intended and unintended) resulting from this interaction [14,15,16]. As a result, the RRR established an initial programme theory (PT): a hypothesised explanation of how a complex intervention or programme is expected to work [17,18]. This initial PT demonstrated that it is essential to establish multidisciplinary teams (C) of competent healthcare providers (HCPs) (C) in order for them to provide person-centred care (M) and involve older people and their informal care giver(s) in the care process (M). This has a positive effect on the functionality of older people (O), hospital-related outcomes (O), and the quality of life of older people (O). Also, by means of a multidisciplinary core team (C) a strong collaboration within and between disciplines can be established (M), which has shown to increase the satisfaction levels of older people, informal caregivers and HCPs (O). Next to efficient use of information technology (C), organisational alignment (C) on all levels, and the provision of sufficient financial resources (C) it is important that that training and education of HCPs (C) in e.g. communication skills takes place, for them to communicate effectively with all involved stakeholders (M). This can result in a delayed placement of the older person in a nursing home (O), reduced use of healthcare services (O), and reduced healthcare costs (O).

### Setting ICPs

The environment plays an important role in the development and implementation of ICPs [19,20,21]. Too often ICPs lack a theoretical underpinning and hence have been accused to jump to solutions and to do more harm than good [22]. What may successfully work in one setting regarding ICPs, may not work in a different setting. Ideally, the appropriate combination of components of an ICP, needs to be developed and based on the values and preferences of the local setting, however this remains underexposed in the literature [19,20,21]. So, notwithstanding the insights derived from the RRR, as mentioned previously, ultimately ICPs need to fit the local setting. The National Health Care Institute in the Netherlands indicated that, ideally, existing, often incidentally developed, local care initiatives should be replaced by conceptualised ICPs for community-dwelling older people. However, different stakeholders in different parts of the Netherlands may hold different beliefs about why, how and for whom an ICP may result in what outcomes and when.

### Study objective

As the RRR provided an international perspective on the items that play an important role in ICPs for older people, but ICPs need to fit the local setting, it is essential to determine which items are relevant for the Dutch setting. Based on that, stakeholders are provided with evidence and practical guidance to establish effective ICPs. This can help to reduce the degree of heterogeneity present in outcomes of ICPs. The aim of this study is to assess consensus on the relevance of context items, mechanisms, programme-activities, and outcomes of ICPs for community-dwelling frail older people for the Dutch setting by various stakeholders and to refine the PT for the Dutch setting, where appropriate.

## Methods

An e-modified Delphi study was conducted to assess consensus for the Dutch setting on the context items, mechanisms, programme-activities and outcomes of ICPs for community-dwelling older people, which emerged from the RRR previously conducted [23]. A Delphi study consists of multiple rounds in which data are collected by sending out a questionnaire that needs to be filled in by a panel of experts on a particular topic. The anonymous responses are aggregated and shared with the panel after each round in the form of a group result [19,24]. In a classical Delphi study, the aim is to elicit opinion and gain consensus, may consist of three or more rounds, and has an open qualitative first round which allows Delphi panel experts to record responses. In this study, the term ‘modified’ refers to a Delphi study that consisted of two rounds, and where in round 1 Delphi panel experts were provided with items of the RRR, of which they are requested to assess their relevance for the Dutch setting [25,26]. A Delphi study is an efficient method for obtaining valuable input from multiple experts in a relatively short timeframe and clarifies which items are more/less relevant and why, or which items are missing from the theory presented, in this case the RRR. Information on consensus among experts is particularly useful in the process of refining the PT and explaining why integrated care does (not) work for (frail) older people, how, and in this specific context.

### Selection of participants

A purposive sampling strategy was used to identify experts with relevant experience in the field of integrated care for older people, aiming for diversity regarding age, gender, profession, and the setting of the ICP(s) they were involved in. In order for the experts to be selected for the Delphi panel, they needed to be actively involved in the implementation of programmes regarding integrated care for (frail) older people at home which were being monitored or evaluated in the Netherlands. Their active involvement in the implementation of ICPs depended on their role as e.g. researchers, healthcare providers, policy advisors, managers etc. Participants for the Delphi expert panel were recruited across the Netherlands through the professional networks of various parties involved in this study, i.e. the commissioner of the current study, a steering committee established for the larger study (see Acknowledgements) and, the researchers of the current study. Experts who met the selection criteria were invited by email with information about the study objectives and details of the Delphi study. Those who gave informed consent were included in the study.

### Delphi round 1

Participants were sent an electronic questionnaire via a weblink (SurveyMonkey). The questionnaire started with an introduction of the study, an explanation of the objectives, the structure of the questionnaire, and the definitions of the constructs: context, mechanisms, programme-activities, and outcomes. The questionnaire continued with six general questions regarding gender, age, highest level of education, current job position, number of years working within the position, and number of years of experience with integrated care for older people. The questionnaire contained another 71 questions related to ICPs [13]. Participants were asked to indicate the relevance of 15 context items, 14 mechanisms, 20 programme-activities and 22 outcomes. Relevance was measured on a 9-point Likert scale (1 = very irrelevant, 9 = very relevant), with scores 1–3 considered as irrelevant, 4–6 as equivocal/ambiguous and, 7–9 as relevant. Context items included e.g. offering training and education to healthcare professionals, and having organisation support and coordination on all levels; mechanisms included e.g. involvement of older people and informal caregivers, and having effective communication between all stakeholders, programme-activities included e.g. performing comprehensive geriatric assessments, and deployment of case management; and outcomes included e.g. delayed move to nursing home, and quality of life (see Appendix A for the complete list). The questionnaire ended with two open questions. In this part, participants were able to provide additions to the context items, mechanisms, programme-activities, and/or outcomes in the questionnaire. The participants were also asked for general comments/suggestions about items and the questionnaire itself. Data collection of round 1 took a total of two weeks.

### Delphi round 2

In the second Delphi round, items on which dissensus was found during the first Delphi, were included. The questionnaire started with the same general questions as round 1. Subsequently, participants were asked to reassess the relevance of the context items, mechanisms, programme-activities, and outcomes on a 9-point Likert scale. At the end of the questionnaire, participants were asked for general comments/suggestions on the items and the questionnaire. During the second round, participants were shown a summary of the group results from the first Delphi round, including 1) the median assessment results and interquartile range (IQR) on each item, 2) the level of (insufficient) consensus between the participants and, 3) whether consensus achieved. The IQR is the difference between the 3^rd^ and 1^st^ quartile in which 50% of core values lie [27]. The IQR also shows the degree of convergence of the answers [28,29,30,31]. A summary of the group results were shown to give insight into the level of (dis)agreement between experts in the first round and to generate additional insights about the specific item(s). It has been shown that providing feedback regarding the level of group agreement reached, influences the achievement of level of consensus subsequently [32]. Data collection of round 2 took a total of two weeks.

### Data analysis

The measures concerning the operationalization of the level of consensus among participants were determined in advance [33]. In the literature, no standard threshold for consensus is offered [34], with thresholds for consensus ranging from 55%–100% [35]. In this study, the 9-point scale was categorized into three ranges: 1–3 as irrelevant; 4–6 as equivocal; and 7–9 as relevant. The cut-off point for consensus among panel members was set on 75% [34,36,37], including the condition that less than 15% of the panel needed to have a scoring in the 1–3 range [38,39]. All items with scores in the 4–6 range and without consensus, were presented again to the expert panel in Delphi round 2. ***Table 1*** demonstrates when an item was defined as irrelevant, equivocal, or relevant based on the overall median panel score in both rounds. The degree of consensus of the respondents on each context item, mechanism, programme-activity, and outcome was analysed based on the median scores of the group. Only fully completed questionnaires in both rounds were included in the analyses. The analyses were performed in MS Excel.

**Table 1:** Rules on consensus and dissensus in different point-ranges.


		OVERALL PANEL MEDIAN IN 1–3 POINT RANGE	OVERALL PANEL MEDIAN IN 4–6 POINT RANGE	OVERALL PANEL MEDIAN IN 7–9 POINT RANGE

**Round 1**	Dissensus (<75%)	Equivocal → included in round 2	Equivocal → included in round 2	Equivocal → included in round 2

	Consensus (≥75%)	Irrelevant	Equivocal → included in round 2	Relevant

**Round 2**	Dissensus (<75%)	Equivocal	Equivocal	Equivocal

	Consensus (≥75%)	Irrelevant	Equivocal	Relevant


### Refined PT

Based on the findings of the Delphi study, the PT presented in the RRR was adjusted where appropriate. Consensus on items being relevant, remained part of the PT or were added to the PT. Consensus on items being irrelevant or no consensus on items were removed from the PT.

### Ethics

As this study does not involve patients or study subjects, according to the Dutch Medical Research in Human Subjects Act (WMO) in the Netherlands, an ethical approval was not needed. However, all participants provided their consent and participation in the survey was anonymous.

## Results

### Participants

**Figure 1:**
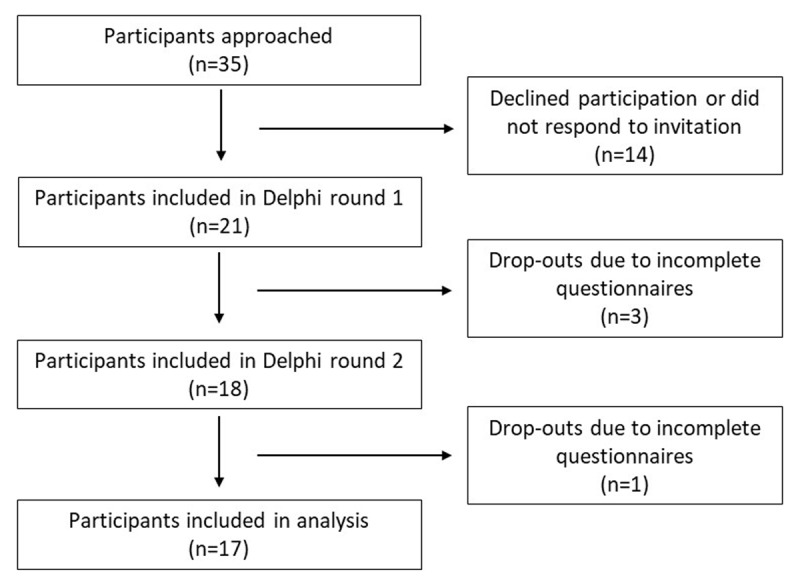
Flowchart of participant inclusion.

A total of 35 people was approached to participate in the Delphi study, of which 21 people agreed (***Figure 1***). One person mentioned she did not have the time to participate, whereas the other 13 did not respond to our invitation and thus did not provide a reason not to participate. Of the 21 participants, three did not fully complete the questionnaire in round one (completion rate = 86%), and one in round two (completion rate = 94%). One participant in round one mentioned she found the questions too hard to interpret. Other participants did not provide a reason for not completing the questionnaire. The final data analyses included responses of 17 participants. In ***Table 2*** the characteristics of the participants are shown.

**Table 2:** Characteristics of the Delphi panel members.


CHARACTERISTIC		PARTICIPANTS (N = 17)

Gender (%)	Female	65

	Male	35

Age (years)	Min-Max	38–61

	Average (SD)	52.5 (7.3)

Highest level of education (%)	Bachelor	12

	Master	47

	PhD	41

Background (%)^#^	Research/academic	24

	Healthcare provider	41

	Other^^^	47

Years of experience	Min-Max	3–35

	Average (SD)	16.4 (10.8)


# Several Delphi panel members had expertise in different backgrounds. ^ ‘Other’ included e.g. policy advisors, IC programme managers/project leaders, lecturers.

### Level of consensus

The results on each of the context items, mechanisms, programme-activities, and outcomes of the first and second Delphi round are shown in Appendix A and B respectively. ***Table 3*** shows the total number of items per category (context, mechanisms, programme-activities and outcomes), the number of items on which consensus was achieved, and how many items were equivocal in both two rounds.

**Table 3:** Results of Delphi round 1 and 2.


CATEGORY	TOTAL NUMBER OF ITEMS	ITEMS FOUND RELEVANT (CONSENSUS), N (%)	ITEMS UNDECIDED (EQUIVOCAL), N (%)

**Round 1**			

Total	71	51 (72)	20 (28)

Context	15	12 (80)	3 (20)

Mechanisms	14	12 (86)	2 (14)

Programme-activities	20	12 (60)	8 (40)

Outcomes	22	15 (68)	7 (32)

**Round 2**			

Total	20	6 (30)	14 (70)

Context	3	2 (67)	1 (33)

Mechanisms	2	1 (50)	1 (50)

Programme-activities	8	2 (25)	6 (75)

Outcomes	7	1 (14)	6 (86)


In the first round, 51 of the total 71 items were considered relevant and consensus was achieved among the experts. The overall median rating by the experts was between the 7–9 point range with a consensus level over 75%. Twenty items remained undecided and were considered equivocal. The panel median for these items was in the 4–6 point range (eight items) and 7–9 point range (12 items) with consensus lower than 75% within the same 3-point region. Consensus on items being found relevant among the experts was observed in the mechanisms (86% of the items), followed by the context items (80% of the items), the outcomes, (68% of the items), and programme-activities (60% of the items). Experts did not propose any additional items to include in round 2. In round 2, two participants indicated that the outcome factor ‘well-being of older person’ was missing in the questionnaire.

In the second round, the 20 items that were rated as equivocal in round one were included. Of these items, consensus on six items was found among the experts. The overall median of the experts was between the 7–9 point range with a consensus level over 75%. For 14 items (70%) the degree of relevance remained undecided (equivocal). The overall median of the experts was in the 4–6 point range (three items) and in the 7–9 point range (11 items). Consensus on items being relevant among the experts was observed for the context items (67% of the items), followed by the mechanisms (50% of the items), programme-activities (25% of the items) and the outcomes (14% of the items). After two rounds, for 57 items of the total 71 items consensus was achieved among the experts whereby all the items were considered relevant.

### Description of items

#### Context items

The context items that were found to be highly relevant for the Netherlands among the Delphi panel experts concern organizational support and coordination, (financial) resources and incentives to invest in integrated care for frail older people, the alignment of existing health and social care systems, and the smart use of (information) technologies. Experts agreed that training of professionals to be competent and highly skilled and having a multidisciplinary core team were context items of great importance. The integration of case management in a broader program or the healthcare system, as well as, offering guidance and support to older people and informal caregivers was also considered relevant. In light of the context item ‘offering remunerative and financial support’, experts mentioned that the financing of integrated care for older people should be led by patients’ needs. This entails that financing should not only be restricted to medical care, but also include social care and support for caregivers.

The context items for which insufficient consensus was found after two rounds concerned the degree of integration of Advanced Practice Nurses (APN) in the healthcare system, for which no reason was provided.

#### Mechanisms

Among the Delphi panel experts there was agreement on the relevance of involving older people and their caregiver(s) (e.g. in shared decision-making, developing care plans) and of the importance of establishing a good relationship between the older person and the HCPs as mechanisms. Communicating effectively plays an essential role, not only between HCPs to ensure optimal interprofessional collaboration, but also between HCPs and older people and their informal caregivers. Enabling collaboration structures were considered very relevant for optimal functioning of a fully integrated interprofessional care team. Also, the HCPs need to provide person-centred care by putting the older person central and focus on the needs, preferences, and possibilities of the individual.

Delphi panellists disagreed on the relevance of focusing on system goals (e.g. improved national system integration) for ICPs. A reason for not finding this mechanism relevant, was not provided.

#### Programme-activities

Experts considered the programme-activities identification and selection of the right target group, incorporating risk prevention in ICPs, performing comprehensive geriatric (home) assessments, and frequent (preventive) home visits to be highly relevant for the Dutch situation. Various care activities, such as the development and implementation of individual care plans, setting up a hospital discharge plan, medication adjustment and alignment (e.g. at care transition) were also considered programme-activities of high relevance. Supporting self-management of older people, the provision of case management, as well as empowerment of patients were found relevant too.

The Delphi rounds also demonstrated that the degree of relevance was undecided for multiple programme-activities, such as the generic and disease-specific deployment of APNs, performing (telephone) follow-up appointments, having specialized clinics regarding memory/dementia care in primary care, standardization of processes, and the use of information technology (IT) for risk inventory and reminders. However, a reason on disagreement was not provided.

#### Outcomes

A high degree of relevance was found for increased functionality, improved self-management of the older person, quality of life (mixed results in the literature), improved (perceived) health, decreased decline in mental health (e.g. depression), a higher satisfaction of the patient, informal caregiver(s) and HCP(s). Moreover, the possibility for the older person to stay longer at home, and hospital-related outcomes (mixed results in the literature) were assessed to be of high relevance. Experts also agreed with high relevance being found on the outcomes of use of hospital services/health system (mixed results in the literature), improved access to healthcare and social care, and improved use of case management services.

Dissensus on relevance has been found concerning the following outcome measures: increase in the performance frequency of early detection screening tests for certain conditions (e.g. diabetes, hypertension, vitamin B12 deficiency) and immunizations (e.g. influenza vaccinations) due to the highly clinical nature of the outcomes; reduced medication use by older people, improved timeliness of communication (e.g. to primary care), cost-effectiveness, and mortality. A reason for disagreement on the latter four outcomes was not found.

#### Refined PT

Based on the findings of the Delphi rounds, the PT was refined for the Dutch setting. In ***Box 1*** this refined PT is shown, with the items that were added, being underlined.

**Box 1 Refined PT on ICPs for community-dwelling frail older people in the Dutch setting.**

C = context; PA = programme-activity; M = mechanism; O = outcome

Considering the increase in the number of older people (C) and decrease in access to hospital beds (C), ICPs with well-designed implementation processes (C) offering continuity of care (PA) are needed. The national and local governments can play a role in facilitating (components of) ICPs by promotion via funding or policy (C) and by providing clarity on legislation and regulations concerning ICPs (C). By means of case finding the right patient population is identified and selected (PA) to deliver the right care at the right time. It is essential to establish well-skilled (C) multidisciplinary teams of competent HCPs (C) providing person-centred care (M) and self-management support (PA) and making sure that patients are empowered (PA) to achieve good health. HCPs need to work closely together (M) and communicate effectively with stakeholders from other domains e.g. primary care, secondary care, community care, social/policy domain, and also informal caregivers (M). By means of education (C) and involving older people and informal caregivers in the care process (M), and trusting the general practitioner (M) and/or the primary HCP (e.g. home visiting professional) (M) a strong relationship between them and the HCP’s (M) should be built. This way management and monitoring of care activities (M, PA) can be optimized with having a clear portfolio of patients (C) whereby continuous feedback to HCP’s (M) needs to be provided. Several programme-activities may contribute to achieving the desired results, such as conducting extensive geriatric assessments/shared assessment processes (PA), setting up individual care plans (PA), having (preventive) home visits (PA), performing case management (PA), managing medication treatment (PA), hospital discharge planning (PA). Next to the alignment of health and social care systems and organizations (C), financial support (C) with e.g. incentives for active participation (M), efficient use of information technology (C), and integration of case management in ICPs (C) emerged also as key elements. ICPs demonstrate positive effects on the functionality (O), mental health (O), self-management skills (O), perceived health (O) of older people, hospital-related outcomes (O), quality of life (O), use of healthcare services including case management (O), and their access to healthcare and social care (O). Besides improved care processes (O), end-of-life discussions were increased (O), the burden on informal caregiver(s) was reduced (O), and there was a delayed placement in a nursing home (O) improving the satisfaction of older people, informal caregivers and HCPs with the care provided (O).

## Discussion

### Principal findings

In this study we aimed to refine the PT for ICPs for community-dwelling frail older people for the Dutch setting by providing insight into the level of consensus on the relevance of context items, mechanisms, programme-activities, and outcomes identified in the RRR. Based on two Delphi rounds, consensus was reached on a set of 57 out of 71 items (80%) of the initial PT, derived from a previous conducted RRR using international literature. Based on the findings of the Delphi study, the initial PT was extended. The added items in the refined PT included increase in the number of older people, decrease in access to hospital beds, well-designed ICP implementation processes, case management, having a clear portfolio of patients, the role of the national/regional governments, aligning existing health- and social care systems, management and monitoring of care activities, strong relationship between older person and HCPs with patients putting their trust in GP, providing continuous feedback to HCPs. These added outcomes were self-management, perceived and mental health, burden on informal caregiver(s), frequency of end-of-life discussions, healthcare access, and care processes. In the refined PT the items ‘having follow-up appointments’ (programme-activity) and ‘healthcare costs/cost-effectiveness’ (outcome) were removed. Also, not finding consensus on the relevance concerning the inclusiveness of APN may illustrate that there is unclarity about the APN role as part of ICPs in the Netherlands. As nowadays primary care assistant practitioners play an important role in primary care for older people, the main role of APNs in ICPs is not fulfilled like before. The main role of APNs seems to have changed over time from practitioner to consultant [12,40].

### Comparison to previous studies

When comparing our findings with those of other studies, it must be noted that there are not many Delphi studies on integrated care specifically for older people. Briggs et al. (2018) generated consensus on the actions required to implement the World Health Organization Integrated Care for Older People (ICOPE) approach [41]. In line with our study, consensus was found on setting up individualised interdisciplinary care plans for patients, active case finding, incorporating prevention programmes, performing geriatric assessments, care delivery by interdisciplinary teams, educational support for formal and informal carers, and the use of data sharing platforms [41]. Items on which no consensus was found by Briggs et al. (2018), were the use of provider report cards, traditional and complementary medicines, and the development of new work cadres [41]. Zonneveld et al. (2020) investigated the values that underpin integrated health services delivery and found consensus on values such as ‘person-centred’, ‘co-produced’, ‘collaborative’, ‘preventative’, and ‘co-ordinated’, comparable to our findings [42]. Regarding values related to IC, no consensus was found on ‘sustainable’, ‘innovative’, ‘proficient’, ‘safe’, and ‘realistic’ due to not being specific or essential enough for IC [42]. They, however, did not focus on programmes for older people specifically.

### Strengths and limitations

The strength of the current study lies in the use of the structured, electronic Delphi technique to further refine the PT in our RRR and explaining why IC does (not) work for (frail) older people, how, and in what context. To the best of our knowledge, we are one of the first to opt for a Delphi study following a RRR, whereas often individual interviews are conducted. Given the scarcity of resources, this appears to be an efficient method for obtaining meaningful input from multiple experts in a relatively short timeframe. This method makes it clear which items are more/less relevant, and/or which items are missing from a RRR and why items are considered less relevant.

However, a few limitations need to be considered for this Delphi study. The first one being the size of the Delphi panel. We invited 35 experts to participate in the Delphi study, but not all responded to our invitation. Nonetheless, sufficient diversity in the Delphi panel was achieved, which is considered more important in terms of validity of study findings. Currently, there are no universally agreed criteria for the selection of experts, and the minimum or maximum number of experts on a panel [34,43]. A second limitation concerns the formulation of questions in the survey. Delphi panel members indicated that several questions were open for own interpretation and could be explained in more detail. The high level of consensus reached after two rounds given a diverse panel, is however very encouraging. To clarify questions in the second Delphi round, some were slightly reformulated or a brief explanation was included. A third limitation relates to the e-interaction between panel members. Exchange of arguments between experts and the authors was only possible digitally, which has hindered in not or partially being able to explain the lack of consensus. In order to acquire more information on the reasoning of members, a blended or ‘physical’ Delphi study could be more suitable.

### Implications for practice and research

The findings of this study can be valuable for both HCPs, policymakers and researchers involved in the development, implementation and/or evaluation of ICPs for older people. Considering the interrelatedness of items, it is suggested to collectively implement the items mentioned in this study, to increase the effectiveness of ICPs. Developing a network in which various stakeholders (e.g. general practitioners, primary care assistant practitioners, pharmacists, community nurses, informal caregivers and older people) have good partnerships, can ensure a better connection between provided services and the needs and preferences of older people. Any forms of consultation to structurally exchange knowledge and expertise may support the (complex) care demand of individual older people. In an ideal situation, tailor-made interventions are offered depending on the different degrees of level of frailty of the older person. In addition, it is important to provide for a systematic risk inventory (e.g. by means of information technology), in which older people at risk are identified in an early stage and subsequently proactive policy can be pursued from the network.

As in this study the context items, mechanisms, programme-activities, and outcomes have been assessed on their relevance, a next step in further research would be to see to what extent these have been implemented and reached the intended outcomes within their context. Also, items on which no consensus was found need to be further investigated on the reason behind it and to explore whether ICPs in the Netherlands are conceptually different than elsewhere or not. Additionally, further validation of context items, mechanisms, programme-activities, and outcomes needs to take place by involving the older people, informal caregivers, and federations for older people/patients [44,45].

## Conclusion

In this study, consensus within the Delphi panel was reached on a set of 57 out of 71 items (80%) based on two Delphi rounds, with items being found relevant. Based on the findings of the Delphi study, the PT for ICPs for older people in the Dutch setting was refined. The added items in the refined PT included increasing number of older people, decreasing access to hospital beds, well-designed ICP implementation processes, case management, having a clear portfolio of patients, the role of the national/regional governments, aligning existing health- and social care systems, management and monitoring of care activities, strong relationship between older person and HCPs with patients putting their trust in GP, providing continuous feedback to HCPs. Further validation of context items, mechanisms, programme-activities, and outcomes needs to take place by involving the older people, informal caregivers, and federations for older people/patients. Additionally, items on which no consensus was found, need to be further investigated on the reason behind it.

## Additional Files

The additional files for this article can be found as follows:

10.5334/ijic.5682.s1 Appendix A. Results Delphi round 1.

10.5334/ijic.5682.s2 Appendix B. Results Delphi round 2.
