# Supplementary material for: Consensus on Integrated Care for Older People Among Dutch Experts: A Delphi Study
Source: Int J Integr Care. 2021 Dec 8;21(4):30. doi: 10.5334/ijic.5682 (PMC8663748; doi:10.5334/ijic.5682)
Supplement: Appendix A. — Results Delphi round 1. [file ijic-21-4-5682-s1.pdf]

Appendix A – Results Delphi round 1

Items	Median and IQR	Consensus in 7-9 range (%)	Consensus in 4-6 range (%)	Consensus in 1-3 range (%)	Overall consensus
Context					
Increase in the number of older people	8 (1)	83	11	6	Relevant
Decrease in access to hospital beds	8 (2)	78	17	6	Relevant
Offering training and education to healthcare professionals (to enhance knowledge and skills)	9 (1)	94	6	0	Relevant
Having a clear portfolio of older people/patients	8 (3)	61	33	6	Equivocal
Having more focus on home visits through promotion by the government (through funding or policy)	6 (2)	39	56	6	Equivocal
Organisational support and coordination on all levels (with the required cultural change)	8 (1)	94	6	0	Relevant
Degree of integration of Advanced Practice Nurses (APN) in the system (organisation of care provision)	8 (2)	61	28	11	Equivocal
Integration of case management in a broader program/the healthcare system	8 (2)	94	0	6	Relevant
Clarity about legislation and regulations among healthcare professionals	8 (1)	89	11	0	Relevant

Aligning existing health and social care systems	9 (0)	100	0	0	Relevant
Offering education, guidance and support to older people and informal caregivers	9 (1)	100	0	0	Relevant
Offering remunerative supports to e.g. invest in a new approach and its management	9 (1)	89	6	6	Relevant
Having a good implementation process of IC programmes	9 (1)	94	6	0	Relevant
Having a multidisciplinary core team (deployment of general practitioner, geriatrician, community nurse, pharmacist, etc.)	9 (2)	89	11	0	Relevant
Using (information) technologies e.g. to facilitate clinical practice, enhance data exchange, improve access to electronic patient files	8 (2)	78	22	0	Relevant
Mechanisms					
Involvement of older people and informal caregiver(s) (e.g. in developing care plans, during hospital discharge, shared decision making etc.)	9 (0)	100	0	0	Relevant
Management and monitoring of care activities (continuity of contact between elderly and health professionals	8 (1)	94	6	0	Relevant
Effective communication between all	9 (0)	100	0	0	Relevant

stakeholders (older people, informal caregiver(s), healthcare professionals of different departments)					
Relationship between older person/informal caregiver(s) and healthcare professionals	9 (1)	100	0	0	Relevant
Availability of emergency telephone services after office hours	8 (2)	89	11	0	Relevant
Home visiting health professional is the designated primary healthcare provider	8 (2)	78	17	6	Relevant
Intensive collaboration of teams within and between different departments and domains with structures of collaboration	8 (1)	94	6	0	Relevant
Continuous feedback to HCP	8 (1)	83	11	6	Relevant
Challenges in mobilizing resources	8 (3)	78	22	0	Relevant
Focus on organisational goals (e.g. cost savings)	8 (2)	78	17	6	Relevant
Focus on system goals (e.g. improved system integration)	8 (2)	72	28	0	Equivocal
Incentives for active participation	7 (3)	56	39	6	Equivocal
Providing person-centred care (focus on the wishes and possibilities of the patient, focus on care process instead of outcomes, confidence in possibilities to improve well-being)	8 (1)	89	11	0	Relevant
Trust that GPs follow recommendations and benefit from	8 (1)	89	11	0	Relevant

collaboration					
Programme-activities					
Multidisciplinary interventions (offering patient health education, exercise programmes, social activities close to home, adjusting medication treatments, and treatment plan)	8 (1)	94	6	0	Relevant
Focus on behavioural change and perception of older person's readiness	9 (2)	89	11	0	Relevant
Standardization of processes	8 (3)	72	22	6	Equivocal
Empowerment of older people	9 (1)	94	6	0	Relevant
Performing comprehensive geriatric (home) assessments (physical, social and psychological)	9 (1)	89	11	0	Relevant
Use of information technology (IT) for risk inventory and reminders	6 (2)	44	50	6	Equivocal
Development and implementation of individual care plans	8 (1)	89	11	0	Relevant
Disease specific deployment of APN's (control of risk factors and complications)	6 (2)	33	50	17	Equivocal
Generic deployment of APN's (improving patient autonomy)	8 (2)	72	22	6	Equivocal
Shared assessment processes	8 (2)	67	33	0	Equivocal
Discharge planning from hospital	9 (2)	89	6	6	Relevant
Performing (telephone) follow-up appointments	8 (2)	67	33	0	Equivocal
Frequent home visits (also for prevention)	8 (1)	78	17	6	Relevant

Supporting self-management of older people	8 (1)	94	6	0	Relevant
Monitoring the patient's care provision during transition e.g. from hospital to home	8 (1)	94	0	6	Relevant
Identification and selection of target group and incorporating prevention in integrated care interventions.	8 (0)	89	11	0	Relevant
Adjusting and aligning medication treatment (e.g. at transition), medication counselling and self management	8 (1)	83	17	0	Relevant
Case management/deployment of case manager	8 (2)	67	33	0	Equivocal
Specialty clinics in primary care (memory/dementia)	6 (1)	17	72	0	Equivocal
Offering continuity by coordination among multiple health professionals	8 (1)	94	0	6	Relevant
Outcomes					
Increase in performance of early detection screening tests for specific diseases	5 (3)	17	61	22	Equivocal
Increase in performance of immunizations	5 (4)	6	67	28	Equivocal
Increased functionality/physical activity level	8 (1)	83	17	0	Relevant
Positive effect on goal attainment, empowerment and satisfaction with care process	8 (2)	94	6	0	Relevant

Improvement in self-management by patients	8 (1)	78	17	6	Relevant
Decreased decline in mental health (e.g. depression)	8 (1)	89	6	6	Relevant
Reduced medication use	7 (2)	61	39	0	Equivocal
Higher satisfaction older person, informal caregiver, and healthcare professional	8 (1)	89	6	6	Relevant
Improved perceived health	8 (1)	89	6	6	Relevant
Reduced burden on informal caregiver(s)	8 (1)	94	6	0	Relevant
Improved use of case management services	5 (2)	28	67	6	Equivocal
Increased frequency of end-of-life discussions	8 (2)	89	11	0	Relevant
Use of hospital/healthcare system (heterogenous effects in literature)	8 (1)	89	6	6	Relevant
Hospital related outcomes (less visits to emergency care, less (re)admissions, less bed days) (heterogenous effects in literature)	8 (1)	94	6	0	Relevant
Delayed move to the nursing home/less time in nursing homes	8 (1)	83	11	6	Relevant
Improved timeliness of communication (e.g. to primary care)	8(2)	72	22	6	Equivocal
Decreased frailty	8 (2)	83	17	0	Relevant
Improved access to healthcare and social care	8 (1)	83	11	6	Relevant
Improved care processes (clinical	8 (0)	89	6	6	Relevant

responsibility across organisations, information sharing, flexible use of resources, interdisciplinary working, shared financial responsibility)					
Healthcare costs/cost-effectiveness (heterogenous effects in literature)	8 (2)	67	28	6	Equivocal
Quality of life (heterogenous effects in literature)	9 (1)	89	6	6	Relevant
Mortality (heterogenous effects in literature)	6 (2)	22	61	17	Equivocal
