# Supplementary material for: Consensus on Integrated Care for Older People Among Dutch Experts: A Delphi Study
Source: Int J Integr Care. 2021 Dec 8;21(4):30. doi: 10.5334/ijic.5682 (PMC8663748; doi:10.5334/ijic.5682)
Supplement: Appendix B. — Results Delphi round 2. [file ijic-21-4-5682-s2.pdf]

Appendix B – Results Delphi round 2

Items	Median and IQR	Consensus in 7-9 range (%)	Consensus in 4-6 range (%)	Consensus in 1-3 range (%)	Overall consensus
Context					
Having a clear portfolio of older people/patients	8 (1)	94	6	0	Relevant
Having more focus on home visits through promotion by the government (through funding or policy)	8 (1)	82	12	6	Relevant
Degree of integration of Advanced Practice Nurses (APN) in the system (organisation of care provision)	7 (3)	71	29	0	Equivocal
Mechanisms					
Focus on system goals (e.g. improved system integration)	7 (2)	59	41	0	Equivocal
Incentives for active participation	8 (1)	82	18	0	Relevant
Programme-activities					
Disease specific deployment of APN's (control of risk factors and complications)	7 (3)	59	29	12	Equivocal
Generic deployment (improving patient autonomy) of APN's	7 (3)	65	29	6	Equivocal
Standardization of processes	7 (2)	65	29	6	Equivocal
Use of information technology (IT) for risk inventory and reminders	7 (2)	71	29	0	Equivocal
Shared assessment processes	7 (1)	82	12	6	Relevant
Performing (telephone) follow-up	7 (4)	59	29	12	Equivocal

appointments					
Case management/deployment of case manager	8 (2)	88	12	0	Relevant
Specialty clinics in primary care (memory/dementia)	7 (3)	53	47	0	Equivocal
Outcomes					
Increase in performance of early detection screening tests for specific diseases	5 (4)	24	47	29	Equivocal
Increase in performance of immunizations	5 (4)	29	41	29	Equivocal
Reduced medication use	7 (3)	71	24	6	Equivocal
Improved timeliness of communication (e.g. to primary care)	8 (3)	71	24	6	Equivocal
Improved use of case management services	7 (1)	82	18	0	Relevant
Healthcare costs/cost-effectiveness (heterogenous effects in literature)	7 (2)	53	47	0	Equivocal
Mortality (heterogenous effects in literature)	6 (3)	41	41	18	Equivocal
